# A Three-Arm Randomized Controlled Trial of Primary One-Anastomosis Gastric Bypass: With FundoRing or Nissen Fundoplications vs. without Fundoplication for the Treatment of Obesity and Gastroesophageal Reflux Disease

**DOI:** 10.3390/medicina60030405

**Published:** 2024-02-27

**Authors:** Oral Ospanov, Nurlan Zharov, Bakhtiyar Yelembayev, Galymjan Duysenov, Irina Volchkova, Kassymkhan Sultanov, Adil Mustafin

**Affiliations:** 1 Department of Surgical Disease and Bariatric Surgery, Astana Medical University, Astana 010000, Kazakhstan; zharov.n@amu.kz (N.Z.); elembaev.b@amu.kz (B.Y.); duysenov.g@amu.kz (G.D.); volchkova.i@amu.kz (I.V.); sultanov.k@amu.kz (K.S.); mustafin.a@amu.kz (A.M.); 2 Surgery Center of Professor Oral Ospanov, Astana 010000, Kazakhstan

**Keywords:** GERD, reflux esophagitis, Nissen fundoplication, obesity, bariatric surgery, one-anastomosis gastric bypass, FundoRingOAGB

## Abstract

*Background and Objectives*: Obesity and gastroesophageal reflux disease (GERD) are steadily increasing world weight and antireflux surgery must be performed simultaneously with bariatric surgery in obese patients. The purpose of this study is to compare bariatric and antireflux results after OAGB with different methods of fundoplication using the excluded stomach and without fundoplication. *Materials and methods*: This open-label, randomized, parallel three-arm trial was conducted from March 2019 and December 2021. All patients underwent laparoscopic one-anastomosis gastric bypass and suture cruroplasty, and then had a follow-up at 24 months. Group 1 of patients had fundoplication FundoRing using the excluded stomach (FundoRingOAGB); Group 2, with Nissen fundoplication using the excluded stomach (NissenOAGB); and Group 3, without fundoplication (OAGB). We studied changes in BMI, GERD symptoms (GERD-HRQL), and the VISICK score. *Results*: Of 219 participants screened, 150 were randomly allocated to 3 groups: FundoRingOAGB group (*n* = 50), NissenOAGB group (*n* = 50), and OAGB group (*n* = 50). At post-treatment month 24, BMI changes were as follows: from 40.7 ± 5.9 (31–53) to 24.3 ± 2.8 (19–29) kg/m^2^ in FundoRingOAGB group; from 39.9 ± 5.3 (32–54) to 26.3 ± 2.9 (23–32) kg/m^2^ in Nissen group; and from 40.9 ± 6.2 (32–56) to 28.5 ± 3.9 (25–34) kg/m^2^ in OAGB group. The mean pre-operative GERD-HRQL heartburn score improved post-op in FundoRingOAGB group from 20.6 ± 2.24 (19.96, 21.23) to 0.44 ± 0.73 (0.23, 0,64); in NissenOAGB group from 21.34 ± 2.43 (20.64, 22.03) to 1.14 ± 1.4 (0.74, 1.53); and in OAGB group 20.5 ± 2.17 (19.9, 21.25) to 2.12 ± 1.36 (1.73, 2.5). GERD-HRQL total scores were from pre-op 25.2 ± 2.7 (24.4, 25.9) to 4.34 ± 1.3 (3.96, 4.7) post-op in FundoRingOAGB group; 24.8 ± 2.93 (24, 25.67) pre-op to 5.42 ± 1.7 (4.9, 5.9) in the NissenOAGB group; and from 21.46 ± 2.7 (20.7, 22.2) to 7.44 ± 2.7 (6.6, 8.2) in the OAGB group. The mean VISICK score improved from 3.64 ± 0.94 (3.7, 3.9) to 1.48 ± 1.26 (1.12, 1.84) in FundoRingOAGB, from 3.42 ± 0.97 (3.1, 3,7) to 2.5 ± 1.46 (2.06, 2.9) in NissenOAGB group and from 3.38 ± 0.88 (3.1, 3,69) to 2.96 ± 1.19 (2.62, 3.2) in OAGB group. *Conclusions*: Antireflux and bariatric results of FundoRingOAGB are better than using the NissenOAGB method and significantly better than OAGB without the use of fundoplication.

## 1. Introduction

The obesity and gastroesophageal reflux disease (GERD) is steadily increasing worldwide. The modified Nissen fundoplication over Roux-en-Y gastric bypass and hiatal hernia repair is the best option for intractable gastroesophageal reflux in patients with obesity [1]. At the same time, gastric bypass is also an effective revision method of surgical treatment in case of unsuccessful antireflux procedures [2]. 

We are one of the first to use a combination of Nissen fundoplication with gastric greater curvature plication for the treatment of GERD and obesity [3]. This method is subsequently referred to as “Nissen plication or Nissen-P” [4]. Unfortunately, plication of the greater curvature of the stomach is inferior in effectiveness to other bariatric procedures and has not been widely used in bariatric practice [5].

Currently, one-anastomosis gastric bypass (OAGB) is a common, simple, and effective bariatric procedure, but excellent metabolic improvement following OAGB remains compromised by the risk of esophageal bile reflux [6,7]. Almost 20% of OAGB reoperations were conversions to RYGB due to bile reflux [8]. Unfortunately, RYGB does not exclude the occurrence of bile and/or acid reflux [9].

Revisional use of the Nissen procedure for the treatment of refractory bile reflux after OAGB may have promise in bariatric surgery [10]. In addition, we developed a modification of the Nissen procedure using the excluded stomach, which we called “FundoRing” [11]. Previously, we published the results of a randomized study using our method FundoRingOAGB (f-OAGB) vs. standard OAGB (s-OAGB) with 24 h pH impedance monitoring data [7]. It has been proven that using FundoRingOAGB to treat obese patients reduces acid reflux esophagitis, significantly more effectively than standard OAGB without fundoplication. This work did not compare the effectiveness of the new FundoRingOAGB method with the well-known Nissen technique, but only compared the results of using FundoRingOAGB and OAGB without fundoplication. Another limitation of the study was the inclusion of patients without GERD symptoms. The special GERD-HRQL questionnaire was not used. We described cases of bile reflux after OAGB without fundoplication that occurred at night when, during sleep, bile entered the trachea, causing episodes of suffocation and fear [7]. Unfortunately, 24 h pH impedance monitoring, unlike GERD-HRQL, does not give data on bile reflux.

The objective of this randomized controlled clinical trial is to compare bariatric and antireflux results after OAGB with different methods of fundoplication using the excluded stomach and without fundoplication.

## 2. Materials and Methods

### 2.1. Trial Design and Ethical Conduct

**Figure 1:**
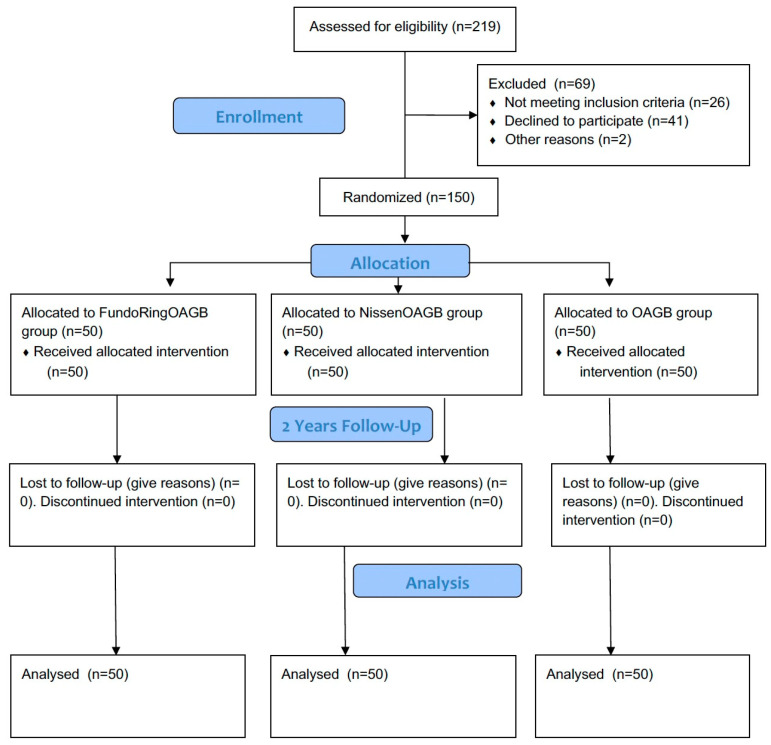
Flow diagram of RCT study.

This single-center open-label, randomized, comparative, clinical, prospective, parallel three-arm groups interventional trial was conducted in a clinic of Astana Medical University. The study protocol was registered as #NCT04828733 in the Clinicaltrials.gov. The CONSORT checklist was used as a guide [12]. The flow diagram of this study is shown in Figure 1.

The protocol of this study was approved on 29 March 2019 by the Ethics Committee and in accordance with the Helsinki Declaration.

### 2.2. Study Settings and Locations

Data were collected at the “Green Clinic” Hospital and the “Surgical Center of Professor Oral Ospanov”, which are clinical sites of the Astana Medical University.

Participant eligibility criteria include the following.

#### 2.2.1. Inclusion

Class I–III obesity (BMI 30.0–50.0 kg/m). Class I Obesity (BMI 30–34.9 kg/m^2^)—when hyperglycemia is inadequately controlled by lifestyle and medical therapy [13].

- Patients with GERD A or B grade of reflux esophagitis (LA grade) after treatment PPI;
- Hiatal hernia (HH) < 5 cm;
- ASA grading 1–2;
- Age 18–60 years old.

#### 2.2.2. Exclusion Criteria

- Giant hiatal hernia (HH) > 5 cm;
- Esophageal shortening;
- Patients with C or D grade of RE (reflux esophagitis);
- Previously surgery on the stomach or esophagus;
- Psychiatric illness.

### 2.3. Intervention Procedures

In the first group, we applied the method of FundoRingOAGB described in the article by Ospanov et al. [11]. In the second group, the method NissenOAGB performed, as described in the articles by Werapitiya, S.B. et al., and Soprani, A. et al. [10,14].

In the third group, a standard OAGB procedure without fundoplication was used by Carbajo M.A. et al. [15]. In either procedure, suture cruroplasty was performed.

**Figure 2:**
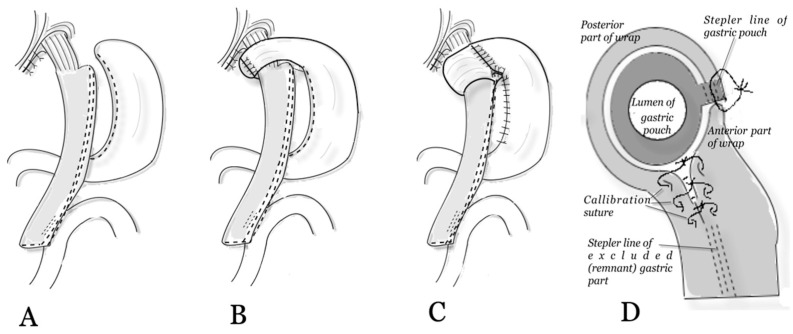
The combination of gastric bypass and fundoplication: (**A**) OAGB without fundoplication (standard). (**B**) NissenOAGB. (**C**) FundoRingOAGB. (**D**) Enlarged view of a fundoplication using the FundoRingOAGB. © 2023 Oral Ospanov.

In Figure 2, the three methods of one-anastomosis gastric bypass in three groups are demonstrated.

The surgical technique of the standard OAGB included the following basic steps (Figure 2A):

1. Staple cutting out stomach for creation of gastric pouch;
2. Measuring the length of the biliopancreatic loop 200 cm;
3. Creation of the gastro-jejunostomy [15];
4. For repair of a hiatal hernia, hiatoplasty (crurorrhaphy) was performed.

In the NissenOAGB group of patients, in addition to the described 4 steps, a Nissen fundoplication was added (Figure 2B) [10,13]. We mobilized the fundus by dissecting the short gastric vessels and suturing the anterior and posterior parts of the fundus at 9–10 o’clock of the “clock face” (Figure 2B).

In the FundoRingOAGB group (Figure 2C), the execution of various steps differed from NissenOAGB [7,11]:

- The gastrosplenic ligament was always divided to ensure good mobilization of the gastric fundus.
- The anterior and posterior parts of the mobilized fundus were joined at 2–3 o’clock. (Figure 2C,D). Circular wrap: wide up to 5–6 cm.
- The next step after circularly fundoplication was creating a partial wrap wide. A continuous nonabsorbable suture of the anterior part of the fundus was sutured to the staple line of the gastric pouch. Partial wrap for additional prevention of slippage of wrap and an increased antireflux mechanism: continuation of the anterior part of the circular fundoplication down along the stapler line of the gastric pouch with stitching to the edge of the stapler suture line for another 2 cm.
- The “FundoRing” is finally created at 5–6 o’clock after double calibration (Figure 2D).
- The suture line was completely covered using a remnant stomach for the prevention of bleeding and a leak of the gastric pouch (Figure 2C).

We calibrated for fundoplication in all cases using a size 32 French bougie.

### 2.4. Sample Size Determination

To calculate the sample size, a standard deviation of 10 was used and a change of 15% in BMI was used as a clinically significant difference. α is set at 0.05—significance level = 5.0%. Effect size [d] was set to 80.0% power. To calculate the sample size, the formula of Brasher and Brant [16] was used: n = 2 (Zα + Z [1 − β])2 × SD2/d2. n is the number of required patients for each sample, Zα = 1.96, Z(1 − β) = 1.28, SD = 10, d = 15. Calculation results: minimum sample size—9.3 patients.

### 2.5. Randomization and Group Allocation

We assessed for eligibility 219 patients fit for OAGB and excluded 69 patients:

- Not meeting inclusion criteria (n = 26);
- Declined to participate (n = 41);
- Other reasons (n = 2).

We distributed (n = 150) into three groups of 50 people each.

Patients were randomized into one of three study groups using the SNOSE method [17]. After opening the envelopes (n = 50 + 50 + 50), the patient and surgeon were aware of the surgical method (without masking). This was an open study.

### 2.6. Study Outcomes

Primary outcome measures:

- Body Mass Index change after 24 months post-op.

Secondary outcome measures:

- Change of GERD and VISICK.

Patients underwent clinical assessment of GERD symptoms (GERD-HRQL), upper endoscopy, and the VISICK score before, and 2 years after OAGB [18,19].

### 2.7. Statistical Methods

For statistical analysis, we used the StatPlus program: MacPro (AnalystSoft Inc., Walnut, CA, USA). Between-group comparisons on quantitative variables were performed using independent one-way analysis of variance. If the ANOVA F test was significant, pairwise comparisons were made using Fisher’s least significant difference (LSD) method. Between-group differences in qualitative variables were assessed using the chi-square test or Fisher’s exact probability test. Statistical significance was set at *p* < 0.05.

## 3. Results

### 3.1. Allocation and Baseline Characteristics

Between March 2019 and December 2021, 150 eligible patients were recruited and randomly allocated to one of three groups: the first (FundoRingOAGB) group (n = 50), the second (NissenOAGB) group (n = 50), and the third group (OAGB without fundoplication) (n = 50). The median follow-up time was 24 months (Table 1).

### 3.2. Primary Outcome

**BMI**

At 24 months post-treatment, BMI changes were as follows: from 40.7 ± 5.9 (31–53) to 24.3 ± 2.8 (19–29) kg/m^2^ in FundoRingOAGB group, from 39.9 ± 5.3 (32–54) to 26.3 ± 2.9 (23–32) kg/m^2^ in NissenOAGB group, and from 40.9 ± 6.2 (32–56) to 28.5 ± 3.9 (25–34) kg/m^2^ in OAGB group.

### 3.3. Secondary Outcome

**GERD-HRQL**

The mean pre-operative GERD-HRQL heartburn score improved post-op (Table 2) in FundoRingOAGB group from 20.6 ± 2.24 (19.96, 21.23) to 0.44 ± 0.73 (0.23, 0,64), in NissenOAGB group from 21.34 ± 2.43 (20.64, 22.03) to 1.14 ± 1.4 (0.74, 1.53), and in OAGB group 20.5 ± 2.17 (19.9, 21.25) to 2.12 ± 1.36 (1.73, 2.5). ANOVA *p*-values (Fisher LSD) are shown in Table 2.

GERD-HRQL total scores were from preop 25.2 ± 2.7 (24.4, 25.9) to 4.34 ± 1.3 (3.96, 4.7) post-op in FundoRingOAGB group, 24.8 ± 2.93 (24, 25.67) pre-op. to 5.42 ± 1.7 (4.9, 5.9) in the NissenOAGB group and from 21.46 ± 2.7 (20.7, 22.2) to 7.44 ± 2.7 (6.6, 8.2) in the OAGB group.

The mean VISICK score improved from 3.64 ± 0.94 (3.7, 3.9) to 1.48 ± 1.26 (1.12, 1.84) in the FundoRingOAGB group, from 3.42 ± 0.97 (3.1, 3,7) to 2.5 ± 1.46 (2.06, 2.9) in NissenOAGB group and from 3.38 ± 0.88 (3.1, 3,69) to 3.3 ± 1.03 (3.00, 3.6) in OAGB group.

These results are illustrated in Table 2.

Pre-operative gastroscopy showed that all patients with GERD had erosive esophagitis (LA grade A or B) and had effective pre-operative treatment with PPI (omeprazole 40 mg/day 2 weeks). We operated on patients only after the healing of erosions in the esophagus and stomach.

The proportion of the size of hiatal hernia in patients is shown in Table 3.

**Average operative time and length of time in hospital**

Average operative time (min) was longer in the FundoRingOAGB group: 93.1 ± 10.7 vs. 89.4 ± 8.7 in the Nissen OAGB group and vs. 78.2 ± 14.3 in the OAGB group (*p* = 0.04) due to additional time required to complete mobilization of the fundus of the excised part of the stomach, followed by the fundoplication and double calibration of fundoplication wrap. There were no differences in length of time in hospital (FundoRingOAGB vs. NissenOAGB vs. OAGB, respectively: 3.3 ± 0.85 vs. 3.2 ± 0.35 vs. 3.4 ± 0.91 days, *p* = 0.88) (Table 1).

**Complications after surgery**

The Clavien–Dindo classification (CDC) was used to classify complications after surgery [20].

The complications of the FundoRingOAGB vs. NissenOAGB vs. standard OAGB through 2 years (CDC grade) are shown in Table 4.

The number of complications (Table 4) was statistically higher in the group where there was no fundoplication: 14 (28%) in the OAGB group versus 8 (16%) cases in the NissenOAGB group. In the FundoRingOAGB group, we did not identify any serious complications. We attribute one case of intra-abdominal bleeding in the NissenOAGB group and the OAGB group to the fact that the stapled suture line was not a hand-sewn reinforcement. As can be seen from Table 4, in contrast to the experimental group, in both control groups, we observed signs of postoperative acid and bile reflux esophagitis. Moreover, in one case in the group of standard OAGB, there was a conversion to RYGB with secondary fundoplication with the use of excluded stomach (FundRing) at 25 months after primary surgery.

We did not observe any other complications, explained by the 15 years of experience of one surgeon who performed all procedures in three groups. Our results prove the safety of a one-anastomosis gastric bypass.

## 4. Discussion

Bile reflux is a common finding in the gastric pouch after one-anastomosis gastric bypass [21]. Interestingly, a study used the Sydney scoring system for bile reflux EGD gastric pouch biopsy, and there is also evidence that 1 year after surgery, there is no difference in bile reflux after OAGB and Roux-en-Y gastric bypass (7.8%), which is considered the gold standard treatment for reflux [22]. Similarly, no statistically significant difference was found in the self-reported history of bile reflux-related symptoms, bile reflux markers in esophagogastroduodenoscopy, and postoperative complications between groups.

In revision bariatric surgery, a combination of fundoplication and gastric bypass is described in a dozen publications [23,24]. Unfortunately, the primary combination of the gastric bypass with fundoplication has rarely been described, and studies are based on small samples. The publication of Soprani et al. reports the results of a retrospective study of 22 patients, with one group of patients treated with the combination of OAGB and Nissen fundoplication [13]. Of the 22 patients, 17 (77.3%) had primary surgery and 5 (22.7%) had Nissen as a revisional surgery after bariatric procedures.

In our opinion, performing primary combined surgery is more rational because of the greater likelihood of complications and technical difficulties accompanying revision surgery in bariatrics. The NissenOAGB surgical technique that we used in the second group is well illustrated in other publications [10,13]. At the same time, the main sequential steps when performing the Nissen procedure for OAGB, as described by other authors, are extrapolated from the surgical technique of Nissen for isolated GERD, not for bariatric aim [25]. This does not take into account the disadvantages of single calibration of wrap and the advantages of using the excluded stomach, which can provide amazing opportunities for reconstructive and plastic surgery during a bariatric procedure. We view the stomach as an ideal living material whose importance was underestimated for use to improve the reliability of stapled sutures, enhance the antireflux function of the esophagogastric junction, and improve bariatric outcomes.

Following our publication, it is known that the FundoRing method differs significantly from OAGB without fundoplication [7]. This publication proves that FundoRingOAGB is better than OAGB in terms of antireflux and bariatric results. The weakness of this study [7] is the lack of comparison of the results of FundoRingOAGB and standard NissenOAGB.

For the first time, our randomized study compares the FundoRing method with Nissen fundoplication and OAGB without fundoplication. According to our results, a single calibration with NissenOAGB at 9–10 o’clock does not create a complete ‘ring’ not only around the upper part of the gastric pouch, but also around the esophagus. Only double calibration at 5–6 o’clock of FundoRingOAGB allows for the creation of an optimal dense and wide “ring” as a sphincter.

As in other studies where fundoplication was not used [26], in our study, bile reflux was detected significantly more often in the OAGB group; in one case a conversion of OAGB to RYGB+ FundoRing was required. We regarded this as a complication grading CDC IIIb [20].

Our results in the NissenOAGB group are like those obtained in the study by Soprani et al. [14], where symptomatic acid GERD was observed in three patients after a similar procedure following surgery, including one de novo and one case of daily biliary reflux. On the contrary, no bile and acid reflux was detected in the FundoRingOAGB group. This indicates that FundoRingOAGB has a better antireflux mechanism vs. NissenOAGB. Moreover, in both of our groups with fundoplications, we did not receive a case of serious dysphagia. It is known that only complete mobilization of the gastric fundus for wrap reduces the incidence of dysphagia [27]. A complication such as thereby twisting the esophagogastric junction is excluded.

At the same time, according to our results, in the group where no fundoplication method was used, bariatric and antireflux results were the worst. However, there is an opinion that Roux-en-Y gastric bypass also effectively treats GERD without fundoplication [28].

Our study showed that performing only OAGB without fundoplication is significantly inferior to OAGB with fundoplication. Noteworthy is the call of some authors to choose a surgical method between fundoplication and RYGB in cases of a combination of obesity and GERD [29]. Moreover, this does not seem logical, because recidivism of GERD after only fundoplication without gastric bypass for obesity patients is most common [30].

We expect good prospects in the future from the use of the FundoRing method for the prevention of recidivism of obesity. But for this, we must perform a study with a long-term group of patients’ follow-up for at least 5 years with FundoRing OAGB.

Despite the longer surgical time in the fundoplication groups, the mean length of hospital stay did not differ in the three groups. This shows that the technical complexity of the surgery did not significantly affect the severity of the postoperative period. The high frequency of detection of hiatal hernia in the three groups, which is associated with the criterion for including patients in this study, deserves special attention. We studied patients with obesity and gastroesophageal reflux disease, which is most often caused by a hiatal hernia, which is often detected only intraoperatively.

Overall, 74% of patients in this study had small hiatal hernias, 23.3% had moderate hiatal hernias, and 2.66% had large hiatal hernias.

Our study had limitations. The study is a single-center trial. A strength of the study was the use of randomization in allocating patients to groups. Despite the lack of masking further actions, randomization made it possible to eliminate the subjective factor during distribution and obtain more objective and reliable research results.

## 5. Conclusions

Antireflux and bariatric results of FundoRingOAGB are better than using the NissenOAGB method and significantly better than OAGB without the use of fundoplication. Continued research and long-term follow-up with the groups are required to recommend a routine use combination of one-anastomosis gastric bypass with fundoplication (FundoRingOAGB).

## Figures and Tables

**Table 1:** The characteristics in FundoRingOAGB group vs. NissenOAGB group vs. OAGB group mean ± SD (range).

Characteristics	FundoRingOAGB Group (n = 50)	NissenOAGB Group (n = 50)	OAGB Group (n = 50)	*p*-Value
Age (years)	40.3 ± 10.3 (20–60)	38.7 ± 9.8 (18–60)	39.2 ± 8.6 (19–53)	0.59
Sex (female/male)	45/5	43/7	44/6	-
Weight (kg)	112.4 ± 19.1 (75–160)	111.6 ± 18.1 (87–154)	113.0 ± 21.0 (78–178)	0.47
Height (cm)	1.7 ± 0.6	1.7 ± 0.9	1.7 ± 0.8	0.9
Mean BMI (kg/m^2^)	40.7 ± 5.9 (31–53)	39.9 ± 5.3 (32–54)	40.9 ± 6.2 (32–56)	0.96
Average operative time of procedures (min)	93.1 ± 10.7	89.4 ± 8.7	78.2 ± 14.3	0.04
Mean length of hospital stay (days)	3.3 ± 0.85	3.2 ± 0.35	3.4 ± 0.91	0.88
Mean BMI (kg/m^2^) at 2 year follow-up	24.3 ± 2.8 (19–29)	26.3 ± 2.9 (23–32)	28.5 ± 3.9 (25–34)	0.04
Change in BMI (kg/m^2^)	16.4	13.6	12.4	-
The median follow-up months (range)	24 (19–31)	24 (18–34)	24 (21–38)	-

At baseline, there were no differences in demographics and body mass index in all three groups (Table 1).

**Table 2:** Score of GERD-HRQL and VISICK (Mean ± SD, 95% CI).

Characteristics	Intervention Assignment			ANOVA *p*-Values (Fisher LSD)			
	FundoRingOAGB Group n = 50	NissenOAGB Group n = 50	OAGB Group n = 50	Overall	1 vs. 2	1 vs. 3	2 vs. 3
GERD-HRQL heartburn score							
Before intervention	20.6 ± 2.24 (19.96, 21.23)	21.34 ± 2.43 (20.64, 22.03)	20.5 ± 2.17 (19.9, 21.25)	0.15	0.1	0.89	0.08
After intervention	0.44 ± 0.73 (0.23, 0,64)	1.14 ± 1.4 (0.74, 1.53)	2.12 ± 1.36 (1.73, 2.5))	0.001	0.004	0.001	0.00008

**Table 3:** The proportion of the size of hiatal hernia in patients.

Groups	Size of Hiatal Hernia (cm)			
	Small HH ≤2	Moderate HH >2–≤4	Large HH >4–≤5	Total
1. FundoRingOAGB (n = 50)	38 (76%)	9 (18%)	3 (6%)	50 (100%)
2. NissenOAGB (n = 50)	36 (72%)	14 (28%)	0	50 (100%)
3. OAGB (n = 50)	37 (74%)	12 (24%)	1 (2%)	50 (100%)
All groups	111 (74%)	35 (23.3%)	4 (2.66%)	150 (100%)

**Table 4:** Complications of the FundoRingOAGB vs. NissenOAGB vs. standard OAGB through 2 years (CDC grade).

Complication	Intervention Assignment			
	FundoRing OAGB Group N = 50	NissenOAGB Group n = 50	OAGB Group n = 50	Overall *p*-Value
Total complications (CDC * grade)	0 (0%)	8 (16%)	14 (28%)	0.045
Bleeding (staple line) (CDC II)	0	1	1	>0.5
Acid reflux esophagitis (CDC I)	0	5	9	>0.5
Total bile reflux esophagitis	0	2	4	0.04
Bile reflux Esophagitis (CDC I)	0	2	3	>0.5
Bile reflux esophagitis conversion OAGBto RYGB+ FundoRing (CDC IIIb)	0	0	1	>0.5

* CDC—The Clavien–Dindo classification.

## Data Availability

All data are available from the corresponding author Oral Ospanov, (bariatric.kz@gmail.com).
